# JNER: a forum to discuss how neuroscience and biomedical engineering are reshaping physical medicine & rehabilitation

**DOI:** 10.1186/1743-0003-1-1

**Published:** 2004-10-13

**Authors:** Paolo Bonato

**Affiliations:** 1 Department of Physical Medicine & Rehabilitation, Harvard Medical School, Spaulding Rehabilitation Hospital, 125 Nashua Street, Boston MA 02114, USA; 2 The Harvard MIT Division of Health Sciences and Technology, Cambridge, MA, USA

## Abstract

Advances in neuroscience and biomedical engineering deeply affect the clinical practice of physical medicine & rehabilitation. New research findings and engineering tools are continuously made available that have the potential of dramatically enhancing the ability of clinicians to design effective rehabilitation interventions. This quickly evolving research field is difficult to track because related literature appears in a wide range of scientific journals. There is a need for a scientific journal that offers to its readership a forum at the intersection of neuroscience, biomedical engineering, and physical medicine & rehabilitation. The Journal of *NeuroEngineering and Rehabilitation* (*JNER) *is intended to fill this gap and foster cross-fertilizations among these disciplines. By making readily available to clinicians selected studies with potential impact on physical medicine & rehabilitation, *JNER *is anticipated to foster the development of novel and more effective rehabilitation strategies. Conversely, by presenting clinical problems to a readership of neuroscientists and engineers, *JNER *is expected to generate innovative work in neuroscience and biomedical engineering with future applications to physical medicine & rehabilitation. *JNER *will leverage on Open Access as a means to guarantee that its content is readily available to scientists, clinicians, and the general public thus promoting scientific and technological advances that are relevant to rehabilitation. *JNER *is an Open Access initiative. Open Access assures dissemination to the widest possible audience and is seen by many as essential for publicly funded research. BioMed Central offers an outstanding platform to make *JNER *possible and allow neuroscientists, biomedical engineers, and clinicians to see their work published in a timely manner and thus make an immediate impact in the field of rehabilitation. *JNER *will focus on innovative work with higher likelihood of a dramatic impact on rehabilitation. Thus, priority will be given to outstanding and visionary scientific reports, i.e. those proposing exceptionally innovative concepts with great potential in the field.

## A new journal for a quickly evolving research field

During the past decade, we have witnessed profound changes in physical medicine & rehabilitation originated by advances in neuroscience and biomedical engineering. For example, imaging and neurological assessment methods have dramatically improved the management of patients with motor impairments; robotics and artificial muscle research have generated revolutionary concepts in orthotics and prosthetics; and advances in cortical recordings and the understanding of central nervous system mechanisms have changed the way clinicians look at movement disorders. These techniques and others have brought about, and will continue to give rise in the future to, dramatic advances in physical medicine & rehabilitation.

As advances in neuroscience and biomedical engineering continue to generate new techniques, with tremendous impact in the field of physical medicine & rehabilitation, it becomes apparent that there is an urgent need for establishing an outlet for the intersection of these three research fields. *Journal of NeuroEngineering and Rehabilitation (JNER) *aims to provide such an outlet, hosting the introduction of new methods and the discussion of their clinical implications, and offering an opportunity to publish, in a timely manner, articles relevant to the cross-fertilization of neuroscience, biomedical engineering, and physical medicine & rehabilitation.

*JNER's *editorial board [1] demonstrates the commitment of the journal to interdisciplinary research and international representation. Members of the editorial board are leading scientists working in different parts of the world in the research areas of neuroscience, biomedical engineering, and physical medicine & rehabilitation. They share an interest in scientific work that has potential impact on clinical practice in physical medicine & rehabilitation and an enthusiasm for Open Access. The editorial board is pleased to become a part of the growing group of institutions and individuals who work to promote Open Access – BioMed Central currently publishes over 100 Open Access journals covering all areas of biology and medicine, and has over 450 institutional members from about 40 countries.

## Open access to advance science and clinical practice

*JNER's *Open Access policy changes the way in which articles are made available to the scientific community. First, all articles become freely and universally accessible online, and so an author's work can be read by anyone at no cost. Second, the authors hold copyright for their work and grant anyone the right to reproduce and disseminate the article, provided that it is correctly cited and no errors are introduced [2]. Third, a copy of the full text of each Open Access article is permanently archived in online repositories separate from the journal. *JNER's *articles are archived in PubMed Central [3], the US National Library of Medicine's full-text repository of life science literature, and also in repositories at the University of Potsdam [4] in Germany, at INIST [5] in France and in e-Depot [6], the National Library of the Netherlands' digital archive of all electronic publications.

Open Access has four broad benefits for science and the general public. First, authors are assured that their work is disseminated to the widest possible audience, given that there are no barriers to access their work. This is accentuated by the authors being free to reproduce and distribute their work, for example by placing it on their institution's website. It has been suggested that free online articles are more highly cited because of their easier availability [7]. Second, the information available to researchers will not be limited by their library's budget, and the widespread availability of articles will enhance literature searching [8]. Third, the results of publicly funded research will be accessible to all taxpayers and not just those with access to a library with a subscription. As such, Open Access could help to increase public interest in, and support of, research. Note that this public accessibility may become a legal requirement in the US if the proposed Public Access to Science Act is made law [9]. Fourth, a country's economy will not influence its scientists' ability to access articles because resource-poor countries (and institutions) will be able to read the same material as wealthier ones (although creating access to the internet is another matter [10]).

Open Access will increasingly become an accepted way to disseminate information to the scientific community and the public at large. By becoming part of the movement for Open Access, *JNER *will contribute to make the latest advances in neuroscience and biomedical engineering, which have the potential to impact on the clinical practice of physical medicine & rehabilitation, readily available to scientists, clinicians, and the general public. Because of its inherent interdisciplinary nature, *JNER *will foster further advances in the field thanks to the cross-fertilization among science, technology, and clinical practice. Science and technology are expected to offer new tools to design clinical interventions and, vice versa, clinical problems are anticipated to foster basic research in neuroscience and the development of new technologies. Besides, increased awareness of the way science and technology can improve clinical outcomes will lead to better quality of healthcare in rehabilitation. Changes currently occurring in this field are so dramatic that we expect, in a few years, that modernized rehabilitation inpatient and outpatient units will be completely different from what is the state-of-the-art today. For instance, we envision that continuous monitoring of patient status will be performed via miniature, wireless, wearable sensors which not only allow clinicians to monitor vital signs, but also track motor activities and provide a means to analyze motor patterns associated with recovery. Furthermore, robotic devices will be used to enhance physical therapy, ad hoc protocols will be designed for each patient, and augmented and virtual reality tools will enhance rehabilitation by becoming part of routine exercise protocols.

## Outstanding and visionary articles make the difference

Open Access to outstanding and visionary scientific reports appears to be a tremendous tool to increase the speed at which clinical practice changes as a result of advances in neuroscience and biomedical engineering. By prioritizing outstanding and visionary publications, *JNER *intends to provide a forum for ideas and concepts that could make a difference in physical medicine & rehabilitation by innovating the design of clinical interventions.

Publication in *JNER *is free for the first 6 months following the launch of the journal. Manuscripts submitted after this period will be subject to an article-processing charge on acceptance. Waiver requests will be considered on a case-by-case basis, by the Editor-in-Chief. Authors can circumvent the charge by getting their institution to become a 'member' of BioMed Central, whereby the annual membership fee covers the article processing charges for authors publishing in any of the BioMed Central journals. Current members include NHS England, the World Health Organization, the US National Institutes of Health, Harvard, Princeton and Yale universities, and all UK universities [11]. No charge is made for articles that are rejected after peer review. Many funding agencies have also realized the importance of Open Access publishing and have specified that their grants may be used directly to pay article-processing charges [12].

The article-processing charges pay for efficient and thorough peer review, for the article to be freely and universally accessible in various formats online, and for the processes required for inclusion in PubMed and archiving in PubMed Central, e-Depot, Potsdam and INIST. Funding available to *JNER's *editorial board will be solely used to further promote the journal and to continuously increase the scientific quality of *JNER's *articles.

The first articles published in the journal demonstrate the commitment of *JNER *to high quality, prioritizing visionary work, and focusing on research that has the potential of a great impact on physical medicine & rehabilitation. Forthcoming articles will further prove such commitment. Topics of interest to come in the next few months are virtual and augmented reality in rehabilitation, wearable technology in rehabilitation, methods for the analysis of movement, and robotics applied to rehabilitation. These are all topics of great relevance for the research at the intersection of neuroscience, biomedical engineering, and physical medicine & rehabilitation.

Special thanks to the authors of the first articles published in *JNER *as well as to the authors who submit their manuscripts in the future and support our journal and the Open Access initiative. Members of the editorial board and reviewers have done some excellent work; special thanks to them and the Managing Editor, Sara Midwood, for their contributions to *JNER*.

## References

[B1] Journal of NeuroEngineering and Rehabilitation, Editorial Board. http://www.jneuroengrehab.com/edboard/.

[B2] BioMed Central Open Access Charter. http://www.biomedcentral.com/info/about/charter.

[B3] PubMed Central. http://www.pubmedcentral.org.

[B4] Potsdam. http://www.uni-potsdam.de/over/homegd.htm.

[B5] INIST. http://www.inist.fr/index_en.php.

[B6] e-Depot. http://www.kb.nl/.

[B7] Lawrence S (2001). Free online availability substantially increases a paper's impact. Nature.

[B8] Velterop J (2003). Should scholarly societies embrace Open Access (or is it the kiss of death)?. Learned Publishing.

[B9] Open Access law introduced. http://www.the-scientist.com/news/20030627/04.

[B10] Tan-Torres Edejer T (2000). Disseminating health information in developing countries: the role of the internet. BMJ.

[B11] BioMed Central Institutional Members. http://www.biomedcentral.com/inst/.

[B12] Which funding agencies explicitly allow direct use of their grants to cover article processing charges?. http://www.human-resources-health.com/info/faq/apc faq.asp?txt_faq_no=8.

